# Electroconvulsive Therapy-Induced Mania in Bipolar Disorder: A Case Report

**DOI:** 10.7759/cureus.75255

**Published:** 2024-12-07

**Authors:** Simon Van Eenoo, Olivier Bollen, Sarah Janssens

**Affiliations:** 1 Psychiatry, Psychiatrisch Ziekenhuis Asster, Sint-Truiden, BEL

**Keywords:** acute mania, adverse effects ect, adverse effects of ect, bipolar disorders, brain and bipolar disorder, ect rare complication, electroconvulsive therapy (ect), lithium, treatment of psychiatric illness and ect

## Abstract

Electroconvulsive therapy (ECT) is widely recognized as a safe and effective intervention for treating severe affective episodes in patients with bipolar disorder. However, it can sometimes precipitate unexpected manic phases in patients treated for a depressive episode, a phenomenon known as ECT-induced mania. While this occurrence is recognized, it remains poorly understood and minimally addressed in the literature. This article presents a case study of a 56-year-old man diagnosed with bipolar I disorder with rapid cycling and late-onset features who received ECT to treat a severe depressive episode with psychotic and catatonic features but developed a manic episode with psychotic elements shortly after ECT initiation. The onset of mania coincided with the discontinuation of valproate, which may have further increased the likelihood of a mood switch.

A literature review suggests four main clinical strategies for managing ECT-induced mania: (1) cessation of ECT, (2) cessation of ECT and initiation of pharmacotherapy, (3) continuation of ECT, and (4) continuation of ECT alongside the addition of lithium as a mood stabilizer. However, the limited and primarily case-based nature of existing research makes it challenging to establish universally applicable treatment guidelines.

In the described case, treatment was modified by temporarily suspending ECT and introducing lithium, which helped stabilize the patient’s manic symptoms. ECT was later resumed with lithium maintenance, achieving improved mood stability. This case highlights the complexities associated with ECT in bipolar disorder, particularly the potential for manic induction, especially when mood stabilizers are withdrawn. It underscores the need for rigorous patient assessment and monitoring, as well as further research to clarify optimal management strategies and preventive measures for ECT-induced mania.

## Introduction

Bipolar disorder is a chronic and severe psychiatric disorder with a substantial impact on patients’ quality of life and a high risk of relapse and is often only diagnosed after several years [1-4]. Treatment modalities include pharmacotherapy, lifestyle adjustments, adjunctive psychotherapy, and electroconvulsive therapy (ECT), the latter of which is especially effective for severe depressive or mixed states that are refractory to medication [5-11]. Although generally well tolerated, ECT can sometimes trigger manic or hypomanic episodes in patients with bipolar disorder, a phenomenon known as ECT-induced mania. Despite its significance, ECT-induced mania remains poorly understood and infrequently addressed in the literature, leaving clinicians with limited guidelines on prevention and management.

This study aims to investigate the risk factors and optimal management strategies for ECT-induced mania in patients with bipolar disorder through the analysis of a case in which a patient experienced ECT-induced mania following valproate discontinuation and a literature review. The objective of this article is to highlight this risk for clinicians, contribute to a deeper understanding of ECT-induced mania, and provide practical insights into managing this complication.

## Case presentation

Patient M., a 56-year-old man, is admitted to the High Intensive Care (HIC) service of the psychiatric hospital for the third time. He was previously admitted to this service on two occasions, each time under compulsory measures. During the initial admission, he was diagnosed with a manic episode within late-onset bipolar disorder after somatic investigations ruled out organicity. A month and a half later, he was discharged on a combination of lorazepam, olanzapine, and valproate.

On the second admission, which followed a few days after the first, an evolving psychotic-depressive state was observed, characterized by fatalistic delusions, self-injurious behavior, and a suicide attempt on the ward. The dosage of lorazepam and valproate was increased (to 4 mg/day and 2000 mg/day, respectively), and sertraline was initiated at 50 mg. The patient demonstrated improvement, and they were discharged from the hospital after a two-month period. 

At the time of the third admission, the patient exhibited symptoms of hypomania, including insomnia and irresponsible financial and social behavior, in addition to depressive elements. He elected to be admitted voluntarily on this occasion. The olanzapine dosage is increased to 30 mg, while the valproate dosage is increased to 2300 mg (blood level control: 81 mg/l). The lorazepam dosage is increased to 4 mg/day, and the sertraline is discontinued. Despite these pharmacological changes, an increase in excitability and suspiciousness is observed, accompanied by an increase in depressive and anxiety symptoms. A mixed episode is identified, with a disorganized psychotic-depressive picture at its core, accompanied by delusions of guilt, catatonia (score of 18 on the Bush-Francis Catatonia Rating Scale), and pronounced suicidal ideation.

In the absence of a favorable response to pharmacological treatment over several weeks, we proceeded to initiate electroconvulsive therapy (ECT). A bitemporal electrode placement was selected, and anesthesia was administered with etomidate and succinylcholine. The patient was then treated with a 30% charge (151.2 mC) on the LOW 0.5 program on the Thymatron System IV. 

The initial two sessions proved inadequate, both in terms of clinical efficacy and as evidenced by the electroencephalogram (EEG). The dosage was increased to 50%, the lorazepam was tapered off, and the valproate was discontinued. At the third session, the desired effect was achieved. 

On the subsequent day, a significant improvement in mood was observed, accompanied by the emergence of pronounced manic features, including megalomania, religious delusions, agitation, disinhibition, flight of ideas, and prolixity. The patient asserts that he has recovered and declined his fourth scheduled course of electroconvulsive therapy (ECT), which was to take place a few days later. We, therefore, decided to suspend the ECT and commence treatment with lithium at a high dose (3x400 mg/day), given its antimanic and mood-stabilizing properties. Additionally, clotiapine is administered 4x/day.

Approximately one week later, the patient is amenable to the notion of undergoing further ECT treatment. Following the resumption of treatment (bitemporal, 50%), a gradual reduction in manic symptoms is observed. After eight adequate sessions, the frequency of ECT is reduced from twice to once a week. 

The patient can be transferred to an open treatment unit in a stable psychiatric condition, having undergone 13 sessions at that point. A few months later, he may be discharged from that unit as well. Lithium is maintained as a long-term medication (400 mg 1.5 times daily). ECT treatment continues on a tapering schedule for six months on an outpatient basis. 

**Figure 1:**
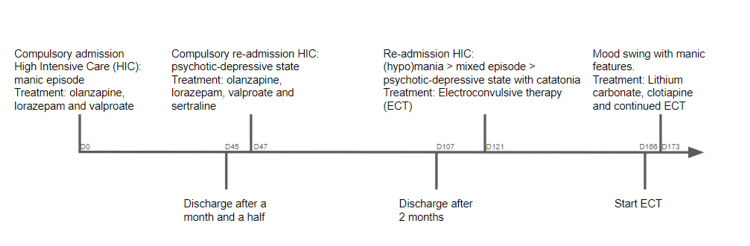
Timeline

A timeline is available in Figure 1.

## Discussion

This case study illustrates the phenomenon of manic switch following electroconvulsive therapy (ECT), underscoring a risk inherent to ECT in bipolar disorder.

While tapering benzodiazepines and mood stabilizers (such as valproate) may improve ECT efficacy, these changes can increase the risk of mood shifts [12]. However, the abrupt and violent onset of the mood change almost immediately after successful ECT suggests that the manic phase was triggered by the ECT treatment while the patient was no longer protected by a mood stabilizer.

Methodology

A comprehensive search of the databases PubMed, Medline, and Embase was conducted to identify relevant articles on the topic. Search strings were constructed using a combination of index terms and variations thereof, focusing on the following key terms: ‘ECT’, 'bipolar disorder', 'electroconvulsive therapy', 'mania', 'ECT-induced mania', and 'lithium'. Additionally, the reference lists of selected articles were searched for any relevant publications. The objective of the literature review was to examine the prevalence of mood reversal, potential explanatory models, and treatment strategies.

Incidence

The prevalence of an ECT-induced switch varies considerably across the literature, with estimates ranging from 0% to 38%. In a 2013 retrospective review, Bost-Baxter et al. examined 105 patients with a history of bipolar disorder (I or II) and reported an incidence of 24.8% for a switch to hypomania or mania [13,14]. However, in 2017, Perugi et al. reported that the risk of ECT-induced mania was 'virtually absent' in a sample of 522 patients [15].

Risk factors

The Bost-Baxter et al. study also examined potential predictors of switching but failed to identify significant characteristics in patients with bipolar disorder or in relation to electrode placement during ECT. The sole predictor that could be identified was the number of ECT sessions in patients not receiving antimanic medication [13].

In naturalistic studies, several variables have been identified as being associated with an increased risk of a switch from depressive to manic mood, both in terms of disease history and current depressive episodes. A younger age or a history of rapid cycles, suicide attempts, and amphetamine use were all associated with an increased risk of a switch from depressive to manic mood. During the current depressive episode, mood elevation, manic symptoms, and comorbid panic attacks have been identified as risk factors [16].

ECT-induced mania can also occur in patients receiving ECT with the diagnosis of unipolar depression, though this appears to be less common [17]. In such cases, ECT-induced (hypo)mania is sufficient to meet the criteria for a diagnosis of bipolar disorder.

Pathophysiology

The underlying biological mechanisms of action of a switch in mood and the role of ECT in this process remain largely unknown. It has been proposed that catecholamine homeostasis may play a role in the naturalistic switch from depressive to manic mood, in addition to disturbances in circadian rhythms (with increased light exposure) [18].

Treatment implications

A systematic literature review conducted by Cloutier et al. in 2021 examined the potential therapeutic approaches for the management of ECT-induced mania [19]. Twelve case reports were included in the review, for a total of 17 patients. Of these patients, eight had a pre-existing diagnosis of bipolar disorder, while the remaining nine were diagnosed with bipolar disorder following the onset of the manic episode. The review concluded that the available data were insufficient to permit the drawing of significant conclusions. However, it did suggest possible guidelines for treatment. The four potential treatment modalities are as follows: (1) cessation of ECT, (2) cessation of ECT and commencement of pharmacological treatment, (3) continuation of ECT, and (4) continuation of ECT in conjunction with lithium.

In some cases, cessation of ECT without additional intervention has been observed to result in the resolution of the manic episode within a few days [20-23]. The majority of patients described (6 out of 7) had no history of bipolar disorder.

Six of the 17 patients described (three of whom had a history of bipolar disorder) were treated by stopping ECT and starting medication. The patients with no history of bipolar disorder were treated with a variety of medications, including quetiapine, valproate, lithium, chlorpromazine, and haldol. In patients with a history of bipolar disorder, lithium was initiated as an antimanic medication, either in combination with other medications or as monotherapy [22,24].

Continuing ECT as an antimanic intervention without the initiation of additional pharmacological treatment also appears to be a viable option. Two case reports, both of patients with a history of bipolar disorder, describe a reduction in manic symptomatology when ECT was continued without alterations to electrode placement [25,26]. A case report from 2022 describes the clearance of manic symptomatology following a change in pulse length and electrode placement from bifrontal to 'left anterior right temporal' (LART) [27].

Additionally, two case reports describe the use of continued ECT in combination with lithium. In both cases, ECT was initially discontinued, but as symptoms persisted, the decision was made to resume ECT in conjunction with lithium [28,29]. These case reports align with the aforementioned case description and, as in the aforementioned case, result in the resolution of symptoms and a stable mood.

The combination of lithium and ECT is a topic of considerable debate in the scientific community. A number of articles have indicated that lithium and ECT can be safely combined. However, other studies have demonstrated an increased risk of delirium and cognitive impairment, including transient anterograde amnesia and prolonged retrograde amnesia [30-35]. The incidence of cognitive impairment and delirium is lower in patients who have received lower doses of lithium, undergone shorter pulse width treatments, and undergone right-unilateral electrode placement. It is hypothesized that the combination of ECT and lithium causes less cognitive impairment and delirium in people with bipolar disorder than in those with unipolar disorder [36]. It is advised that lithium should not be administered the evening before ECT and that one should monitor for early signs of delirium. The combination of lithium and ECT in individuals with bipolar disorder may prevent the onset of ECT-induced mania [23].

Lithium should be continued following the resolution of the affective episode to prevent relapse [37,38]. The combination of ECT and psychopharmaceuticals as maintenance therapy appears to be more effective than psychopharmaceuticals alone in preventing relapse into depression [39].

## Conclusions

Electroconvulsive therapy (ECT) remains a safe and efficacious treatment for patients with bipolar disorder across the spectrum of the disorder's phases. However, it can precipitate a (hypo)manic mood swing. This case report highlights the risk of ECT-induced mania in patients with bipolar disorder, underscoring the need for careful monitoring and individualized treatment. The phenomenon is relatively frequent and clinically relevant, yet it remains poorly understood and infrequently addressed in the literature. The current literature is insufficient to define evidence-based guidelines for the treatment of ECT-induced (hypo)mania. However, some suggestions can be posited. The patient's history appears to be a significant factor in this regard. In patients with no history of bipolar disorder, the cessation of ECT and observation of the subsequent course of events seems a reasonable course of action. In the majority of cases described, this results in spontaneous resolution. If symptoms persist, antimanic therapy (by means of antipsychotics, lithium, or ECT) is indicated. 

In patients with a history of bipolar disorder, a more definitive course of action would be more appropriate. Options here include the commencement of pharmacological treatment (preferably with lithium), the continuation of ECT, or a combination of the two. It is recommended that in patients with a history of ECT-induced mania (or a history of bipolar disorder), lithium should be initiated prior to ECT to prevent ECT-induced (hypo)mania. A significant portion of the available studies are case reports (some of which are relatively dated), and there is still considerable uncertainty regarding the prevalence and pathophysiological mechanisms. Consequently, further research is required with a focus on understanding the incidence, risk factors, and mechanisms associated with ECT-induced mania and on developing evidence-based guidelines to inform clinical practice. Randomized controlled trials investigating preventive strategies, including the pre-ECT use of lithium, would be valuable in establishing robust clinical guidance.
